# Adherence to clinical practice guidelines for using electroconvulsive therapy in elderly depressive patients

**DOI:** 10.1186/s12888-024-05933-7

**Published:** 2024-07-03

**Authors:** Antoine Yrondi, Olivier Blanc, Loic Anguill, Christophe Arbus, Ludivine Boudieu, Marie-Camille Patoz, Adeline Arnould, Thomas Charpeaud, Jean-Baptiste Genty, Racan Abidine, Maximilien Redon, Romain Rey, Bruno Aouizerate, Djamila Bennabi, Wissam El-Hage, Bruno Etain, Jérôme Holtzmann, Marion Leboyer, Fanny Molière, Raphaelle Marie Richieri, Florian Stéphan, Guillaume Vaiva, Anne Sauvaget, Emmanuel Poulet, Emmanuel Haffen, Philippe Courtet, Philippe Fossati, Pierre-Michel Llorca, Ludovic Samalin

**Affiliations:** 1 French Society for Biological Psychiatry and Neuropsychopharmacology, Saint Germain en Laye, France; 2 Service de Psychiatrie Et de Psychologie Médicale, Centre Expert Dépression Résistante FondaMental, CHU de Toulouse, Hôpital Purpan, ToNIC Toulouse NeuroImaging Centre, Université de Toulouse, INSERM, UPS, 330 Avenue de Grande Bretagne, 31059 Toulouse, France; 3 Department of Psychiatry, CHU Clermont-Ferrand, University of Clermont Auvergne, CNRS, Clermont Auvergne INP, Institut Pascal (UMR 6602), Clermont-Ferrand, France; 4 Clinique du Grand Pré, 63830 Durtol, France; 5 Université Paris-Est, UMR_S955, UPEC, Créteil, France Inserm, U955, Equipe 15 Psychiatrie Génétique, Créteil, France AP-HP, Hôpital H. Mondor-A. Chenevier, Pôle de Psychiatrie, Créteil, France Fondation FondaMental, Fondation de Cooperation Scientifique, Créteil, France; 6 INSERM U1028, CNRS UMR5292, University Lyon 1, Lyon Neuroscience Research Centre, Psychiatric Disorders: From Resistance to Response ΨR2 Team, Centre Hospitalier Le Vinatier, Bron, France; 7 Pôle de Psychiatrie Générale Et Universitaire, Centre de Référence Régional Des Pathologies Anxieuses Et de La Dépression, Centre Expert Dépression Résistante FondaMental, CH Charles Perrens, Bordeaux, Laboratoire Nutrition Et Neurobiologie Intégrée (UMR INRAE 1286), Université de Bordeaux, Bordeaux, France; 8 Université de Franche-Comté, UMR INSERM 1322 LINC, Service de Psychiatrie de L’adulte, CIC-1431 INSERM, CHU de Besançon, F-25030, Besançon, France; 9 Clinique Psychiatrique Universitaire, Centre Expert Dépression Résistante FondaMental, CHRU de Tours, Tours, France; 10 Département de Psychiatrie Et de Médecine Addictologique, Lariboisière-Fernand Widal GHU APHP Nord Université Paris Cité Paris, Paris, France; 11 Service de Psychiatrie de L’adulte, CS 10217, Centre Expert Dépression Résistante FondaMental, CHU de Grenoble, Hôpital Nord, Grenoble, France; 12 Department of Emergency Psychiatry and Acute Care, CHU Montpellier, INSERM U1061, Montpellier University, Montpellier, France; 13 Pôle Psychiatrie, Centre Expert Dépression Résistante FondaMental, CHU La Conception, Marseille, France; 14 Service Hospitalo-Universitaire de Psychiatrie Générale Et de Réhabilitation Psycho Sociale 29G01 Et 29G02, Centre Expert Depression Résistante FondaMental, EA 7479, CHRU de Brest, Hôpital de Bohars, Brest, France; 15 University Lille, Inserm, CHU Lille, U1172 - LilNCog - Lille Neuroscience & Cognition & Centre National de Ressources Et Résilience Pour Les Psychotraumatismes (Cn2r Lille Paris), F-59000 Lille, France; 16 Nantes Université, CHU Nantes, Movement, Interactions, Performance, MIP, UR 4334, 44000 Nantes, France; 17 French Society for Biological Psychiatry and Neuropsychopharmacology, STEP Section (Stimulation Transcrânienne en Psychiatrie), Saint-Germain-en-Laye, France; 18 Psychiatric Emergency Service, Hospices Civils de Lyon, F-69005 Lyon, France; 19 France Centre Hospitalier Le Vinatier, Bron; INSERM, U1028; CNRS, UMR5292; Lyon Neuroscience Research Center, PSYR2 Team, F-69000 Lyon, France; 20 University Lyon 1, Villeurbanne, F-69000 Villeurbanne, France; 21 Control-Interoception Attention Team, Paris Brain Institute (ICM), Sorbonne University, INSERM, CNRS, APHP, Hôpital de La Pitié-Salpêtrière, DMU Neuroscience, Paris, France

**Keywords:** ECT, Mood disorder, Major depressive disorder, Bipolar disorder, Geriatric patients

## Abstract

**Objectives:**

Electroconvulsive therapy (ECT) is one of the most effective treatments in mood disorders, mainly in major depressive episode (MDE) in the context of either unipolar (MDD) or bipolar disorder (BD). However, ECT remains a neglected and underused treatment. Older people are at high risk patients for the development of adverse drug reactions. In this context, we sought to determine the duration of MDEs and the number of lines of treatment before the initiation of ECT in patients aged 65 years or over according to the presence or absence of first-line indications for using ECT from international guidelines.

**Methods:**

In this multicenter, retrospective study including patients aged 65 years or over with MDEs in MDD or BD who have been treated with ECT for MDEs, data on the duration of MDEs and the number of lines of treatment received before ECT were collected. The reasons for using ECT, specifically first-line indications (suicidality, urgency, presence of catatonic and psychotic features, previous ECT response, patient preference) were recorded. Statistical comparisons between groups used standard statistical tests.

**Results:**

We identified 335 patients. The mean duration of MDEs before ECT was about 9 months. It was significantly shorter in BD than in MDD- about 7 and 10 months, respectively. The co-occurrence of chronic medical disease increased the duration before ECT in the MDD group. The presence of first-line indications for using ECT from guidelines did not reduce the duration of MDEs before ECT, except where there was a previous response to ECT. The first-line indications reduced the number of lines of treatment before starting ECT.

**Conclusion:**

Even if ECT seems to be a key treatment in the elderly population due to its efficacity and safety for MDEs, the delay before this treatment is still too long.

**Supplementary Information:**

The online version contains supplementary material available at 10.1186/s12888-024-05933-7.

## Key points

• The mean duration of depression before ECT was 8.8 months.

• The duration before ECT for Bipolar Disorder (vs Major Depressive Disorder) is shorter.

• The co-occurrence of chronic medical disease increased the duration before ECT in the MDD group.

• Focusing on the first-line indications for using ECT, only a previous response reduces the duration of depression before ECT.

**Supplementary Information:**

The online version contains supplementary material available at 10.1186/s12888-024-05933-7.

## Introduction

Electroconvulsive therapy (ECT) is one of most effective treatments in mood disorders, mainly in major depressive episode (MDE) in the context of either unipolar (MDD) or bipolar disorder (BD) [1, 2]. It is now well established that ECT is efficient and safe in the elderly population [3]. Moreover, a recent meta-analysis highlighted that ECT was particularly effective in elderly people with moderate and severe MDE [4, 5]. However, ECT remains a neglected and underused treatment [6–8]. In the elderly population, concerns regarding the side effects, mainly cognitive effects, may reduce its use [3]. Nevertheless, ECT should not be considered a "last line" treatment for the elderly population with MDE. It has been shown that its use is safe, with minimal side effects, when appropriate considerations are taken for this population [9].

Moreover, clinical guidelines do not limit the use of ECT for treatment-resistant depression (TRD) [10–12]. Indeed, ECT could be used as a first-line treatment when rapid clinical improvement is required, such as in the presence of psychotic or catatonic features, urgency (e.g. poor oral intake), high suicidality, or when patient preference or previous response to ECT are reported [10–13].

Recently, we highlighted in adult patients with MDE that, despite these recommendations, the mean duration of MDE before ECT was around 10 months and the mean number of lines of treatment before ECT was over 3 [14]. We also showed that the duration of MDE before ECT was longer for a single episode than for recurrent MDD and BD. Moreover, despite the fact that the presence of first-line indication for using ECT was associated with shorter duration of MDE and lower number of therapeutic sequences before ECT, this duration remained over 9 months and the number of lines of treatment over 3 [14].

Because of polypharmacy, multi-morbidity, and age-related changes in pharmacokinetics and pharmacodynamics, older people are high-risk patients for the development of adverse drug reactions (ADRs) [15]. Also, a problem can be the risk of drug interactions with other drugs taken due to chronic medical conditions [16]. Moreover, elderly patients with depression have a higher risk of suicide and more rapid life-threatening outcome. As a consequence, the use of ECT should occur earlier than in the younger population. However, to our knowledge, there is no study assessing delay before accessing ECT in this population.

In this context, we sought to determine the duration of MDEs and the number of lines of treatment before the initiation of ECT in patients aged 65 years or over depending on the presence or absence of first-line indications for using ECT from international guidelines.

## Methods

### Study design and recruiting characteristics

This multicenter, retrospective, cross-sectional study included patients recruited by the French national network of 12 experts [FondaMental Foundation (www.fondation-fondamental.org)] [17, 18] and collaborative centres hosted by academic departments of psychiatry (Besançon, Brest, Clermont-Ferrand, Créteil, Grenoble, Lyon, Marseille, Montpellier, Nantes, Paris, Toulouse, Tours).

### Ethics approval and consent to participate

The assessment protocol was approved by the relevant ethical review board (CPP Sud-Est VI, 2016 / CE 07). This a retrospective study. All data were collected anonymously.

### Population

This study of 335 individuals included all patients over 65 years with MDD or BD who have received ECT as acute treatment for an MDE according to DSM-IV-TR criteria from 1 January 2009 to 1 January 2014. Exclusion criteria were schizophrenia or schizoaffective disorder and autistic spectrum diagnosis (according to DSM-IV-TR criteria).

### Measures

As previously described [14], the following data were extracted from the computerised or paper clinical records: sociodemographic data (age, sex), number of previous MDEs, lifetime diagnosis (MDD single episode, recurrent MDD, BD type 1 and 2), substance use disorders (tobacco, alcohol, cannabis, opioids, psychostimulants) and chronic physical disease comorbidities (cardiovascular, respiratory, neurologic, neoplastic, infectious, systemic, endocrinal, and metabolic). Duration of MDE (delay between the onset of the episode and the first day of ECT) and the number of lines of treatment received consecutively (in monotherapy or in combination) before the initiation of ECT were collected. The reasons for using ECT as previously described, specifically first-line indications, based on international guidelines, were also recorded rating retrospectively the charts of the 335 patients [10–13]. We chose the first line indications as suggested in Canadian [12] and Australian / New Zealand [11, 13] guidelines and confirmed in a review focusing on different mood disorder guidelines [10].

### Statistical analysis

Sociodemographic and clinical data are presented as the mean (SD) for continuous variables and frequency distribution for categorical variables (n, %).

Statistical comparisons between groups of patients were done by using standard statistical tests: the Chi-square test for categorical variables and Student t test, Anova test or Mann–Whitney test, depending on the number and distribution of continuous variables. The distribution was tested with Kernel estimation (Supplementary Fig. 1 and 2).

Analyses were conducted using SAS 9.3 (SAS Statistical Institute, Cary, North Carolina). All statistical tests were 2-tailed, with the α level set at 0.05.

## Results

We identified 335 patients aged 65 year or over who commenced ECT for treatment of MDE (MDD or BD) according to DSM-IV-TR criteria. Age, sex and clinical characteristics are presented in Table 1.

**Table 1:** Age, gender and clinical characteristics of the sample

**Characteristics**		**Mean (SD) or n (%)**			
		Age [65–93]			
		**Total**	**MDD**	**BD**	***p*-value**
Age		74.3 (6.5)	74.6 (6.4)	73.7 (6.5)	NS
Gender	Males	100/335 (29.9)	63/219 (28.8)	37/116 (31.9)	NS
Previous MDE	Mean (SD)	1.47 (0.7)	1.32 (0.8)	1.84 (0.4)	< 0.001
	0	46/289 (15.9)	45 /203 (22.2)	1/86 (1.2)	< 0.001
	1	60/289 (20.8)	48 /203 (23.6)	12/86 (13.9)	
	> = 2	183/289 (63.3)	110 /203 (54.2)	73/86 (84.9)	
*MDE characteristics*	Psychotic features	182/335 (54.3)	121/219 (55.3)	61/116 (52.6)	NS
	Catatonic features	21/335 (6.3)	11/219 (5.0)	10/116 (8.6)	NS
	Melancholic features	241/335 (71.9)	159/219 (72.6)	82/116 (70.7)	NS
	Atypical features	11/335 (3.3)	6/219 (2.7)	5/116 (4.3)	NS
	No features	62/335 (18.5)	43/219 (19.6)	19/116 (16.4)	NS
*Comorbidities*	Chronic medical disease	249/332 (75.0)	164/217 (75.6)	85/115 (73.9)	NS
	Substance use disorder				
	Tobacco	16/318 (5.0)	8/210 (3.8)	8/108 (7.4)	NS
	Alcohol	11/249 (4.4)	4/219 (1.8)	7/116 (6.0)	0.040
	Cannabis, opioid, psychostimulant	6/335 (1.8)	5/219 (2.3)	1/116 (0.9)	NS

*BD* Bipolar Disorder, *MDD* Major depressive disorder, *MDE* Major depressive episode, *NS* Non-significant, *SD* Standard deviation

Values are given as mean (SD) or as n (%) of patients

The mean duration of MDE before ECT was 8.88 (12.03) months. We found a significant shorter duration of MDE in patients with BD compared to MDD (*p* = 0.005) (Table 2). In addition, it seemed that the occurrence of single MDE in MDD increases the duration of MDE before ECT (mean duration = 13.75 months, SD = 13.77) in comparison with recurrent MDE (mean duration = 8.67 months, SD = 11.00) or BD (mean duration = 6.90, SD = 8.88; F = 7.4213, *p* < 0.001). The previous number of MDEs reduces the duration before ECT in the whole sample (*p* < 0.001) and mainly, in the MDD group (*p* < 0.01). Moreover, the co-occurrence of other chronic medical diseases increased the duration before ECT in the MDD group (Table 2).

**Table 2:** Duration (months) of MDE before ECT according to lifetime diagnosis, number of previous MDE, MDE features and comorbidities

		**Mean (SD) or n (%)**								
		Age [65–93]								
		**Total**			**MDD**			**BD**		***p*-value**
***Lifetime diagnosis***		8.88 (12.03)			9.92 (11.91)			6.90 (8.88)		0.005
				***p*-value**			***p*-value**			***p*-value**
**Previous MDE**	0	15.42 (14.24)		< 0.001	15.72 (14.26)		< 0.01	2.00 (-)^a^		NS
	1	10.05 (13.06)			9.43 (11.53)			12.54 (18.38)		
	` ≥ 2	7.41 (9.17)			8.07 (10.41)			6.40 (6.84)		
		**Absent**	**Present**	***p*-value**	**Absent**	**Present**	***p*-value**	**Absent**	**Present**	***p*-value**
***MDE features***	Psychotic features	8.77 (11.12)	8.96 (10.99)	NS	9.64 (12.57)	10.14 (11.39)	NS	7.21 (7.77)	6.63 (9.84)	NS
	Catatonic features	9.00 (11.22)	7.00 (7.85)	NS	10.16 (12.08)	5.29 (6.68)	NS	6.71 (8.90)	8.90 (8.94)	NS
	Melancholic features	8.93 (11.69)	8.85 (10.80)	NS	10.67 (13.52)	9.64 (11.28)	NS	5.86 (6.56)	7.33 (9.69)	NS
	Atypical features	8.89 (11.11)	8.36 (8.86)	NS	9.82 (12.00)	13.67 (9.04)	NS	7.12 (9.02)	2.00 (1.00)	NS
	No features	8.69 (10.51)	9.68 (13.16)	NS	9.50 (11.02)	11.63(15.05)	NS	7.22 (9.41)	5.29 (5.40)	NS
***Comorbidities***	Chronic medical disease	7.14 (9.18)	9.54 (11.59)	NS	7.55 (10.40)	10.93 (12.37)	0.03	6.40 (6.60)	7.12 (9.63)	NS
	Substance use disorder	8.83 (10.91)	11.33 (18.05)	NS	9.85 (11.73)	12.90 (19.71)	NS	6.93 (8.92)	3.5 (-)^a^	NS

*BD* Bipolar Disorder, *MDD* Major depressive disorder, *NS* Non-significant, *SD* Standard deviation

Values are given as mean (SD)

^a^Only one observation

**Fig. 1:**
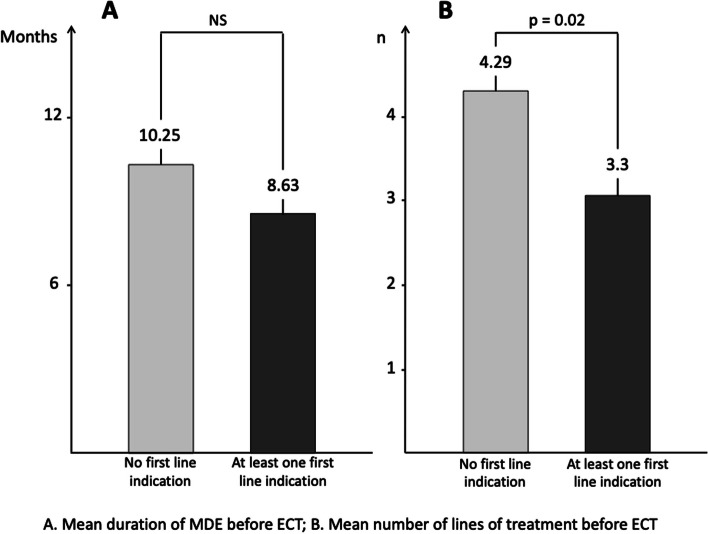
Comparisons according to the presence or absence of at least one first line indications for using ECT in guidelines

The presence of first-line indications for using ECT from guidelines did not reduce the duration of MDE before ECT (Table 3), except for previous response to ECT (*p* < 0.001). However, the first-line indications reduced the number of lines of treatment before starting ECT (Fig. 1).

**Table 3:** Comparisons according to the presence or absence of first line indications for using ECT in guidelines

First line indications for using ECT	Presence of first line indications n (%)	Mean duration of episode before ECT (months)		
		**Absent**	**Present**	***p*-value**
High suicidality	34/335 (10.1)	8.84 (10.93)	9.11 (12.12)	NS
Urgency	142/335 (42.4)	9.74 (11.48)	7.70 (10.33)	NS
Previous ECT response	104/335 (31.0)	10.40 (12.02)	5.49 (7.46)	< 0.001
Catatonic features	21/335 (6.3)	9.00 (11.22)	7.00 (7.85)	NS
Psychotic features	182/335 (54.3)	8.77 (11.12)	8.96 (10.99)	NS
Patient preference	14/335 (4.2)	8.88 (11.05)	8.73 (11.19)	NS

Values are given as mean (SD) or n (%)

*SD* Standard deviation

## Discussion

To our knowledge, this is the first study focusing on the adherence to guidelines for using ECT in the elderly population focusing on the duration of MDE and the number of treatments before ECT. In this population, the mean duration of MDEs before ECT was 9 months. This duration is in line with data on adult patients with MDE [14]. Unlike this study, the presence of first-line indications for using ECT from guidelines, such as the presence of psychotic or catatonic features, urgency (e.g., poor oral intake), high suicidality, or patient preference did not reduce the delay before ECT in our elderly population. However, a previous response to ECT reduced the delay before ECT. The lack of difference between the presence and the absence of first-line indications for using ECT, in elderly population, could be explained by the fact that the delay in case of the absence of the first line indications seems shorter in this population compared to the adult population [14].

However, these first-line indications reduced the number of treatments tested before ECT.

Unexpectedly, the delay before ECT was significantly longer for MDD patients with comorbid chronic physical diseases. This could be explained, in part, by psychiatrists’, anaesthetists’, and geriatric practitioners’ concerns about the safety of ECT in these patients. The management of these physical comorbidities may also have delayed the management of MDE in hospitals providing ECT.

Although there is an important delay before management of BD after the onset of the disease [19], we highlighted a difference focusing on the delay before ECT between MDD and BD. In our elderly population, it seems that once correctly diagnosed, patients with BD access ECT more quickly than patients with MDD. Moreover, it seems that patients with recurrent episodes benefit from ECT earlier than those with a single episode. Indeed, the occurrence of previous MDE reduces the duration before ECT. However, we did not find these results in the BD group, probably due to the small number of patients in our cohort with a late life onset episode (only one). Therefore, it seems difficult to interpret these analyses. Better knowledge of the disorder and, in case of a similar episode, a lack of the need for further examination (in order to search for a neurological disorder such as dementia) could explain this difference.

However, the reasons for the long delay before ECT are more difficult to explain in the elderly population except by three points: i) the uncertain or negative attitudes of patients towards ECT [20], ii) the choice/practice of the psychiatrist, and mainly, the difficulties of changing prescription habits despite recommendations, whatever the type of treatment [21] and iii) the difficulties of access to this therapeutic strategy [22, 23] and the lack of relevant training of health professionals [22]. Indeed, a recent meta-analysis showed that old age was a predictor of response and remission focusing on ECT [5]. Moreover, despite the stigma, it is now well established that ECT is safe and well tolerated, especially in the elderly population [24]. It is a safe alternative to pharmacotherapy (which can generate side effects leading to early discontinuation) [24]. Moreover, ECT does not worsen the course of dementia, and is indicated for comorbid depression and agitation in dementia [3]. Therefore, ECT should be used more quickly, mainly in this population, because MDE can lead to death by suicide [25], particularly in the elderly population and it is the first cause of functional disability and poor quality of life [26, 27].

There are some limitations to this work. First, not all health care facilities practicing ECT in France participated in this study. This could limit the extrapolation of results. Second, this is a retrospective study in which data may potentially be missing from medical files. Moreover, it is difficult to assess urgency criteria retrospectively.

## Conclusion

Even if ECT seems to be a key treatment in the elderly population due to its efficacy and safety for MDEs, the delay before this treatment might be too long, probably due to factors such as the capacity for ECT that is very sparse in France and the uncertain or negative attitudes of patients towards ECT. MDEs can, firstly, lead to death by suicide, particularly in the elderly population and, secondly, it is the first cause of functional disability and poor quality of life. Therefore, improving knowledge of the practice of ECT of psychiatrists and facilitating accessibility to ECT, especially in the elderly population, should represent a major public health concern.

### Supplementary Information

Supplementary Material 1.

Supplementary Material 2.

## Data Availability

The datasets used and analyzed during the current study are available from the corresponding author on reasonable request.
