# Supplementary figures and images for: Adherence to clinical practice guidelines for using electroconvulsive therapy in elderly depressive patients
Source: BMC Psychiatry. 2024 Jul 3;24:487. doi: 10.1186/s12888-024-05933-7 (PMC11223384; doi:10.1186/s12888-024-05933-7)

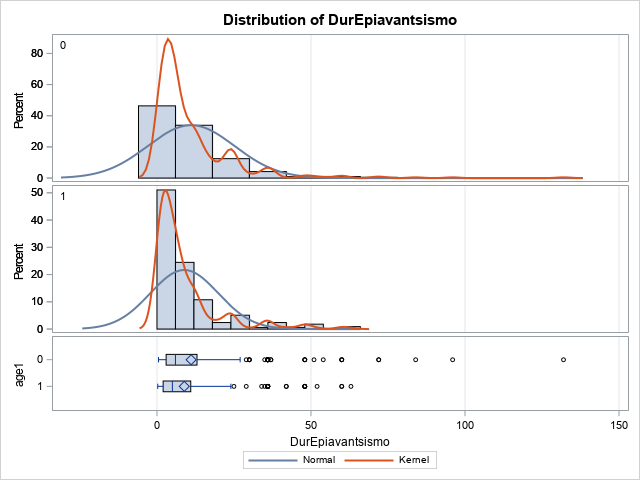


Fig.S1 Duration of episode before ECT

Supplement: Supplementary file 1 — Supplementary Material 1. [file 12888_2024_5933_MOESM1_ESM.docx]

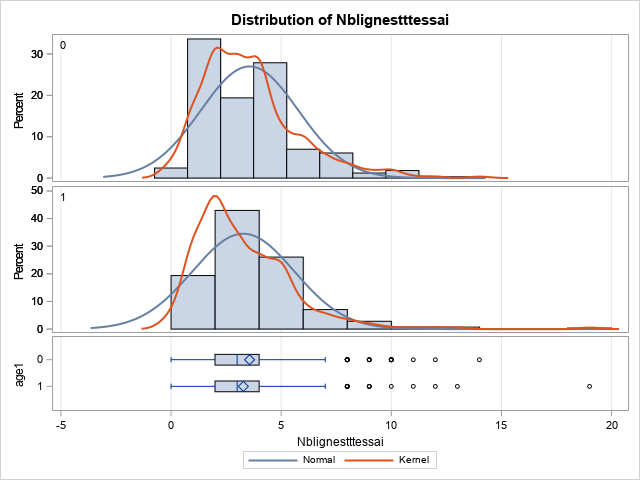


Fig. S2 Number of treatment before ECT

Supplement: Supplementary file 2 — Supplementary Material 2. [file 12888_2024_5933_MOESM2_ESM.docx]
